# Perineural dexamethasone effectively prolongs anaesthesic block duration in total hip arthroplasty, reduces opioid consumption, and does not compromise motor function, nerve integrity, or glycaemic control

**DOI:** 10.1007/s00264-025-06578-1

**Published:** 2025-06-11

**Authors:** Tomasz Reysner, Grzegorz Kowalski, Aleksander Mularski, Monika Pyszczorska, Monika Grochowicka, Anna Perek, Przemyslaw Daroszewski, Malgorzata Reysner

**Affiliations:** 1 Poznan University of Medical Sciences, Poznań, Poland; 2 University of Zielona Góra, Zielona Góra, Poland

**Keywords:** Perineural dexamethasone, Total hip arthroplasty, PENG block, Opioid-sparing analgesia, Motor function preservation, Block duration prolongation

## Abstract

**Background:**

Adequate postoperative analgesia is critical for elderly patients undergoing total hip arthroplasty (THA). The pericapsular nerve group (PENG) block relieves pain while preserving motor function, but its limited duration necessitates adjuncts. This study evaluates the efficacy of perineural dexamethasone in prolonging PENG block analgesia in geriatric THA patients.

**Methods:**

In this double-blinded, randomized controlled trial, 60 patients (≥ 65 years) undergoing THA under spinal anaesthesia were assigned to the PENG group – PENG block with 20 mL 0.2% ropivacaine and the PENG + DEX group – PENG block with 20 mL 0.2% ropivacaine + 4 mg perineural dexamethasone. The primary outcome was time to first rescue opioid administration. The secondary outcomes included total opioid consumption, pain scores (NRS), quadriceps strength, and adverse effects over 48 h.

**Results:**

Dexamethasone significantly prolonged analgesia (16.0 ± 1.3 vs. 9.0 ± 1.7 h, *p* < 0.0001) and reduced opioid use (0.9 ± 1.2 vs. 2.1 ± 1.4 mEQ, *p* = 0.0003). Pain scores were lower at six, 12, and 24 h (*p* < 0.05). Quadriceps strength remained intact in both groups. No nerve injuries were observed (*p* > 0.9999). Blood glucose levels at 12, 24, and 48 h showed no significant differences between groups (*p* > 0.05).

**Conclusions:**

Perineural dexamethasone effectively prolongs PENG block duration, reduces opioid consumption, and does not compromise motor function, nerve integrity, or glycaemic control. It is a promising strategy for optimizing pain control in elderly THA patients.

## Introduction

Total hip arthroplasty (THA) is a widely performed surgical procedure for patients with end-stage hip osteoarthritis, providing significant pain relief and functional improvement [1]. However, postoperative pain management remains a critical challenge, particularly in elderly patients undergoing THA under spinal anaesthesia. Optimal pain control in this population is essential for early mobilization, reduced opioid consumption, and enhanced recovery outcomes [2].

Regional anaesthesia techniques, including the Pericapsular Nerve Group (PENG) block, have gained popularity as part of multimodal analgesia strategies for THA. The PENG block effectively targets the sensory branches of the femoral and obturator nerves, providing potent pain relief while preserving motor function [3]. Several studies have demonstrated its efficacy in reducing postoperative pain scores and opioid requirements when incorporated into enhanced recovery after surgery (ERAS) protocols [4].

Despite its proven analgesic benefits, the duration of the PENG block remains a limiting factor in prolonged postoperative pain control [5]. Perineural dexamethasone, a corticosteroid known for its anti-inflammatory and analgesic properties, has been proposed as an effective adjunct to prolong regional anaesthesia effects [6]. Prior studies in regional anaesthesia have shown that dexamethasone can extend the duration of nerve blocks, thereby delaying the time to first opioid administration and reducing total opioid consumption [7]. However, its efficacy and safety in the PENG block for THA patients remain insufficiently studied.

This double-blind, randomized controlled trial (RCT) aims to evaluate the analgesic efficacy of the PENG block with and without perineural dexamethasone in elderly patients undergoing THA. The study explicitly investigates the time to first rescue opioid administration, total opioid consumption, postoperative pain scores, quadriceps muscle function, and potential adverse effects such as hyperglycaemia or nerve injury. By addressing these outcomes, this study seeks to provide robust evidence on dexamethasone’s role in enhancing the PENG block’s clinical utility, thereby optimizing perioperative pain management in THA patients.

## Methods

### Study design

This double-blinded, prospective, randomized controlled trial (RCT) was conducted at a single orthopaedic centre in Poland. The Bioethics Committee of Poznan University of Medical Sciences approved the study protocol on September 13, 2023 (protocol number 541/2023). Subsequently, the trial was registered at ClinicalTrials.gov (NCT06470139) on August 13, 2024. Before participation, written informed consent was obtained from all patients following ethical research standards. Patient enrollment occurred between August 14, 2024, and January 31, 2025. The study was conducted in full compliance with the principles outlined in the Declaration of Helsinki**.**

### Participants

Patients scheduled for total hip arthroplasty under spinal anesthesia were considered for enrollment before surgery. Inclusion criteria encompassed individuals aged 65 to 100 years who could provide informed consent and reliably report symptoms to the research team.

Exclusion criteria included patients who declined participation or could not provide first-party consent due to cognitive impairment or language barriers.

### Randomization and blinding procedures

A computer-generated randomization process assigned patients in a 1:1 ratio to one of the following groups: the PENG group – received an ultrasound-guided PENG block, and the PENG + DEX group –received an ultrasound-guided PENG block with Dexamethasone. The randomization list was generated using the nQuery Advisor program (Statistical Solutions, Boston, MA, USA) by a researcher who was not otherwise involved in the study. Group allocations were concealed in opaque, sequentially numbered envelopes to ensure allocation concealment until the moment of intervention.

### Blinding protocol

This study maintained double-blinding through strict role separation among researchers. A designated independent researcher, who had no role in patient care or outcome assessment, prepared the randomization list and sealed the group assignments in numbered envelopes. Before the orthopaedic procedure, the"first"consultant anesthesiologist received and opened the assigned envelope and administered the PENG block, or PENG block, with Dexamethasone according to the allocated group. Immediately after performing the nerve block, the"first"anesthesiologist was replaced by a"second"consultant anesthesiologist, who remained blinded to the group assignment and supervised all anesthesia-related procedures during surgery. Throughout the study, the anaesthesia team, surgical team, operating room staff, patients, and outcome assessors remained blinded to the intervention received. Group allocation was only revealed after the completion of statistical analyses to prevent bias.

### Procedures

In both study groups, patients received an individualized dose of midazolam (ranging from 2.5 mg to 7.5 mg p.o.) approximately 30 min before surgery as part of a multimodal preemptive analgesia protocol. The exact dose was tailored based on the patient’s age, weight, and comorbidities. All patients underwent standardized spinal anaesthesia under mild sedation, tailored for geriatric tolerance. Sedation was achieved with a continuous propofol infusion, adjusted within a 2–5 mg/kg/hour range, administered throughout the surgery. The infusion rate was carefully titrated to maintain adequate sedation while minimizing respiratory depression. Oxygen supplementation was provided via a face mask at a flow rate of 3–5 L/min, adjusted according to the patient’s oxygenation status and respiratory function. Spontaneous ventilation was maintained throughout. Spinal anaesthesia was performed at the L3/4 interspace using a 27 G, 90 mm Sprotte needle (PAJUNK) with 4 ml of 0.5% ropivacaine. The surgeon performed no periarticular infiltration during the procedure. The “first” and “second” anaesthesiologists involved in this study had over five years of post-specialty clinical experience focusing on regional anaesthesia, specifically nerve blocks.

#### PENG block procedure

Following the administration of spinal anaesthesia and before the surgical incision, the PENG block was performed with the patient in the supine position. A curvilinear, low-frequency (4–8 MHz) ultrasound probe guided a 22-gauge needle (Stimuplex Ultra 360, 80 mm) inserted in a lateromedial direction. The needle was positioned laterally to the iliopsoas tendon to prevent quadriceps weakness between the ilio-pubic eminence and the anteroinferior iliac spine. Hydro-location was confirmed with 0.5 ml of 0.9% isotonic saline. After confirming negative aspiration, either 20 ml of 0.2% ropivacaine with 2 ml of 0.9% NaCl, or 20 ml of 0.2% ropivacaine with 4 mg of dexamethasone was slowly administered laterally to the iliopsoas tendon, according to the patient’s randomized group assignment.

### Surgical and postoperative protocol

All patients underwent hip arthroplasty under spinal anaesthesia performed by a single surgical team consisting of four orthopaedic surgeons at the Orthopedic Hospital, Poznan University of Medical Sciences. The Direct Superior Approach (DSA) was consistently used across all patients, and an uncemented implant (Smith & Nephew Polarstem/R3) was implanted in each case.

### Postoperative care and follow-Up

A radiographic evaluation was conducted on the first postoperative day. Patients followed a standardized rehabilitation and pain management protocol (detailed in the following sections). Weight-bearing as tolerated was encouraged, and patients were mobilized with hikers. Each patient underwent a minimum of two days of active follow-up postoperatively. An independent researcher collected Primary and secondary outcomes during in-hospital visits, ensuring unbiased data collection.

### Postoperative analgesia and thromboprophylaxis management

Postoperative pain management followed a multimodal analgesic protocol, incorporating a combination of non-opioid and opioid analgesics to optimize pain control while minimizing opioid consumption. The regimen included: Acetaminophen 1.0 g every six h**,** Metamizole 1.0 g every six h**,** Ibuprofen 400 mg every eight h. For rescue analgesia, if a patient’s Numerical Rating Scale (NRS) score reached ≥ 4, a 5 mg intravenous oxycodone bolus was administered. To ensure standardized reporting and facilitate comparisons with other studies, total opioid consumption was expressed in morphine milliequivalents (mEQ), using a standard conversion ratio of 1 mg oxycodone = 1.5 mg morphine.

### Thromboprophylaxis and early mobilization

All patients received daily enoxaparin for four weeks postoperatively as prophylaxis against thromboembolic events. Early mobilization was initiated ten h after surgery, with patients ambulating under supervision using a hiker to promote functional recovery and reduce postoperative complications.

### Outcome measures

The *primary outcome* was the time to the first administration of rescue opioid analgesia, which was assessed by residents from the postoperative and orthopaedic wards who were not involved in the study.

*Secondary outcomes* included total opioid consumption, which was recorded in milligrams of oxycodone from the orthopaedic ward records. This data was converted into morphine milliequivalents (mEQ) using the standardized conversion ratio (1 mg oxycodone = 1.5 mg morphine) to ensure consistency with other studies. Residents and fellows, who were blinded to the study, performed these assessments. Pain intensity was evaluated using the Numerical Rating Scale (NRS) at predefined postoperative time points (4, 6, 12, and 24 h after surgery). The NRS ranged from 0 (no pain) to 10 (worst pain imaginable). Two independent physicians conducted these evaluations, and the final pain score was determined through mutual agreement at the end of the assessment. Quadriceps muscle strength was assessed using the Medical Research Council (MRC) Scale for Muscle Strength, where Grade 5 represents normal muscle strength, Grade 4 indicates movement against gravity and resistance, Grade 3 indicates movement against gravity over nearly the entire range, Grade 2 indicates movement of the limb but not against gravity, Grade 1 represents visible contraction without movement of the limb (not applicable to hip flexion), and Grade 0 indicates no visible contraction. Two independent physicians evaluated quadriceps strength, and the final score was determined by consensus at the end of the assessment.

Nerve injury was assessed retrospectively on the day of discharge based on documented neurological deficits in the orthopaedic ward records. Nerve deficits were classified as follows: 0: No nerve damage, 1: Minor sensory paraesthesia, 2: Complete sensory anaesthesia, 3: Complete motor deficit with or without sensory paraesthesia, 4: Complex Regional Pain Syndrome. Two researchers, blinded to group allocation, reviewed and classified these outcomes.

Blood glucose levels were measured at 12, 24, and 48 h postoperatively. Blood samples were collected by nursing staff, blinded to the study, and analyzed by two independent researchers who were also blinded to group allocation.

### Statistical analysis

The sample size was based on our primary hypothesis that the time to first rescue opioid analgesia would be significantly longer in the PENG + DEX group than that in the PENG group. Our null hypothesis was that there would be no significant difference in the time to first rescue analgesia between these two groups. The time to first rescue opioid analgesia was the primary variable. Based on a pilot study on ten patients, not included in the final analysis, the time to first rescue opioid analgesia was 9.46 ± 4.43 h (mean ± SD) in the PENG group and 14.26 ± 4.16 h (mean ± SD) in the PENG + DEX group. Using pairwise comparison, we calculated the sample size required to detect a difference in the time to first rescue opioid analgesia among the two groups. Accordingly, 22 subjects were required in each group to achieve a statistical power of 95% at a p-value of < 0.05. To facilitate block randomization and account for loss to follow-up, 30 patients per group (60 in total) were recruited.

Statistical analysis was performed using GraphPad Prism 10.1.1 (270) software (GraphPad Software Inc., San Diego, CA, USA). The parametric distribution of numerical variables was evaluated using the Shapiro–Wilk normality test. Differences between groups were assessed using the ANOVA with post hoc Tukey’s test. Categorical variables were compared with the Kruskal–Wallis test, and contingency analysis between groups was conducted using Fisher’s exact test. A p-value of < 0.05 was considered statistically significant and was calculated with 95% confidence intervals (Cl).

## Results

**Fig. 1:**
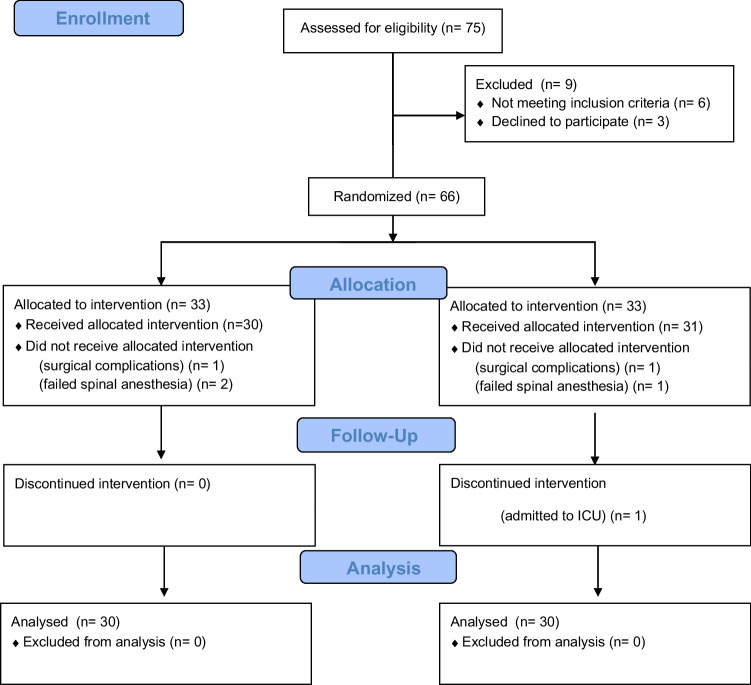
Consort-2010-flow-diagram

As shown in Fig. 1, 75 patients were assessed for eligibility. Nine were excluded, six did not meet the inclusion criteria, and three declined participation. The remaining 66 patients were randomized into two intervention groups: PENG (n = 33) and PENG + DEX (n = 33)**.** In the PENG group**,** 30 patients received the allocated intervention, while three patients did not receive the intervention due to surgical complications (n = 1) and failed spinal anaesthesia (n = 2). Similarly, in the PENG + DEX group**,** 31 patients received the allocated intervention, while two patients did not receive it due to surgical complications (n = 1) and failed spinal anaesthesia (n = 1). During the follow-up period, one patient in the PENG + DEX group discontinued the intervention due to ICU admission. No other patients were lost to follow-up in either group. At the end of the study, 30 patients from each group were analyzed, with no patients excluded from the final analysis. No clinically relevant differences were observed among the group characteristics, as shown in Table 1.

**Table 1:** Baseline characteristics

	PENG	PENG + DEX	*p*
ASA			
ASA II	10	8	0.7787
ASA III	20	22	
Age (years)	70.5 (3.3)	71.0 (2.9)	0.4443
M/F	14/16	15/15	> 0.9999
BMI	29.2 (2.8)	29.8 (2.9)	0.4233
Time of surgery (min)	77.5 (8.2)	77.2 (7.4)	0.9136

*PENG* Pericapsular nerve group, *DEX* Dexamethasone, *BMI* Body Mass Index, *M* male, *F* female, *min* minutes. *p*-value compares the PENG group to the PENG + DEX group

### Primary outcome

**Fig. 2:**
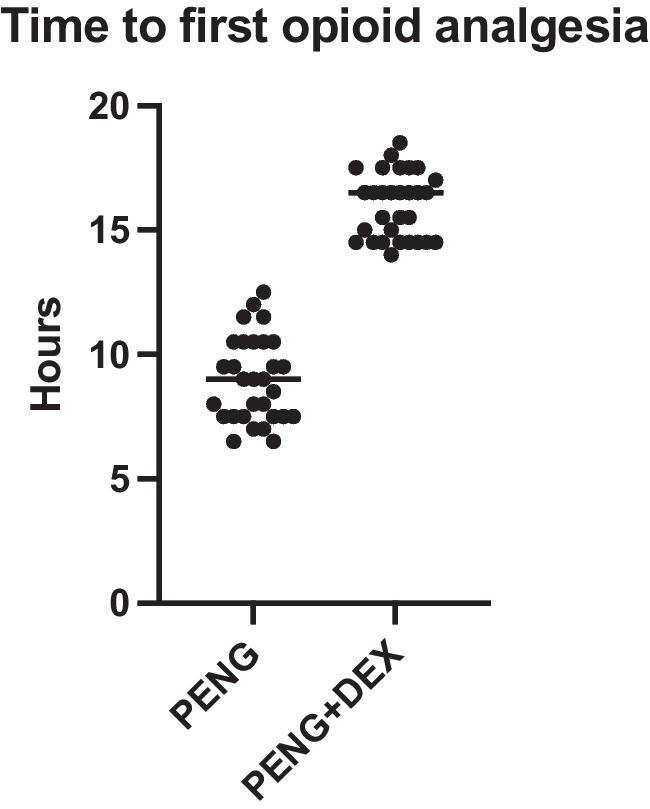
Time to first opioid analgesia

The time to first rescue opioid analgesia was significantly prolonged in the PENG + DEX group compared to the PENG group, as presented in Table 2 and in Fig. 2. Patients in the PENG + DEX group required their first rescue analgesia at a mean of 16.0 ± 1.3 h, whereas patients in the PENG group required rescue analgesia significantly earlier, at 9.0 ± 1.7 h (*p* < 0.0001). The mean difference between the groups was 7.5 h (95% CI: 6.0 to 8.0 h).

**Table 2:** Primary and secondary outcomes

	PENG	PENG + DEX	*p*	Mean difference (95%Cl)
Time to firs rescue opioid analgesia (h)	9.0 (1.7)	16.0 (1.3)	< 0.0001	7.5 (6.0 to 8.0)
48 h opioid consumption (morphine mEQ)	2.1 (1.4)	0.9 (1.2)	0.0003	−2.5 (−2.2 to 0.0)
Postoperative opioid consumption				
Yes	22 (73%)	12 (40%)	0.0182	
No	8 (27%)	18 (60%)		
NRS				
4 h	1.4 (0.6)	1.4 (0.8)	0.8267	0.5 (0.0 to 0.0)
6 h	2.3 (0.9)	1.2 (0.9)	< 0.0001	−0.5 (−2.0 to 0.0)
12 h	2.1 (0.9)	1.6 (0.5)	0.0105	0.0 (−1.0 to 0.0)
24 h	1.8 (0.5)	1.4 (0.5)	0.0064	−1.0 (−1.0 to 0.0)
Quadriceps muscle strength				
Knee extension				
4 h	5.0 (0)	5.0 (0)	> 0.9999	0 (0 to 0)
8 h	5.0 (0)	5.0 (0)	> 0.9999	0 (0 to 0)
12 h	5.0 (0)	5.0 (0)	> 0.9999	0 (0 to 0)
24 h	5.0 (0)	5.0 (0)	> 0.9999	0 (0 to 0)
Hip adduction				
4 h	5.0 (0)	5.0 (0)	> 0.9999	0 (0 to 0)
8 h	5.0 (0)	5.0 (0)	> 0.9999	0 (0 to 0)
12 h	5.0 (0)	5.0 (0)	> 0.9999	0 (0 to 0)
24 h	5.0 (0)	5.0 (0)	> 0.9999	0 (0 to 0)
Nerve damage (0–4)	0 (0)	0 (0)	> 0.9999	0 (0 to 0)
Blood glucose				
12 h	116.9 (16.4)	117.0 (17.5)	0.9678	2.0 (−8.0 to 8.0)
24 h	120.8 (20.2)	115.6 (17.6)	0.2900	−8.5 (−16.0 to 4.0)
48 h	114.1 (14.3)	116.7 (17.5)	0.6406	1.5 (−6.0 to 10.0)

*PENG* Pericapsular nerve group, *DEX* Dexamethasone, *mEQ* milliequivalents, *h* hours. *p*-value compares the PENG group to the PENG + DEX group

### Secondary outcomes

**Fig. 3:**
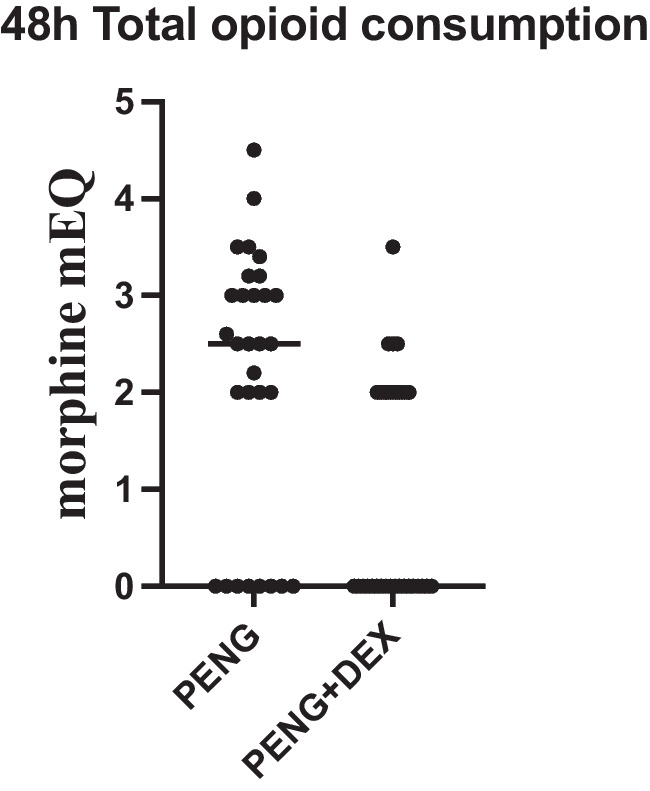
48 h Total opioid consumption

**Fig. 4:**
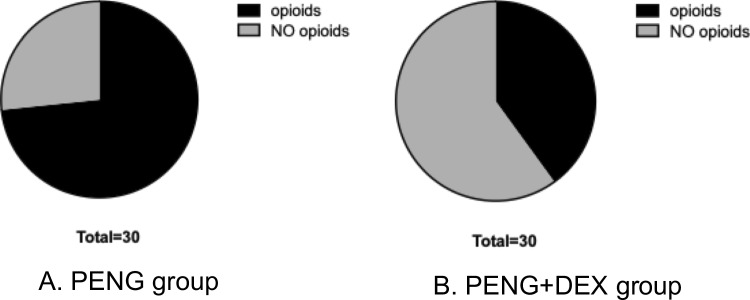
Postoperative opioid consumption

The total opioid consumption over 48 h, expressed in morphine milliequivalents (mEQ), was significantly lower in the PENG + DEX group compared to the PENG group (0.9 ± 1.2 mEQ vs. 2.1 ± 1.4 mEQ, *p* = 0.0003), as seen in Fig. 3. The mean difference was −2.5 mEQ (95% CI: −2.2 to 0.0), demonstrating a clinically significant reduction in postoperative opioid requirements in the PENG + DEX group. Furthermore, the proportion of patients requiring rescue opioid analgesia was significantly lower in the PENG + DEX group (40%) compared to the PENG group (73%, *p* = 0.0182), as seen in Fig. 4.

Postoperative pain scores (NRS) were significantly lower in the PENG + DEX group at multiple postoperative time points: At six h postoperatively, the mean NRS score in the PENG + DEX group was significantly lower (1.2 ± 0.9) compared to the PENG group (2.3 ± 0.9, *p* < 0.0001), with a mean difference of −0.5 (95% CI: −2.0 to 0.0). At 12 h, pain scores remained lower in the PENG + DEX group (1.6 ± 0.5) compared to the PENG group (2.1 ± 0.9, *p* = 0.0105). At 24 h, the PENG + DEX group continued to exhibit significantly lower pain scores (1.4 ± 0.5) compared to the PENG group (1.8 ± 0.5, *p* = 0.0064). At four h postoperatively, pain scores were comparable between groups (*p* = 0.8267).

Quadriceps muscle strength, evaluated using the Medical Research Council (MRC) Scale, remained unchanged between groups at all assessed time points (4 h, 8 h, 12 h, 24 h). Both knee extension and hip adduction consistently maintained a score of 5.0 in all patients, with p > 0.9999 across all time points. This indicates that perineural dexamethasone did not induce any detectable motor weakness or functional impairment.

No cases of nerve injury were observed in either group throughout the study (p > 0.9999), further supporting the safety profile of both interventions.

Postoperative blood glucose levels remained comparable between groups at 12 h, 24 h, and 48 h, with no statistically significant differences: 12 h: PENG + DEX: 116.9 ± 16.4 vs. PENG: 117.0 ± 17.5 (*p* = 0.9678), 24 h: PENG + DEX: 120.8 ± 20.2 vs. PENG: 115.6 ± 17.6 (*p* = 0.2900) and 48 h: PENG + DEX: 114.1 ± 14.3 vs. PENG: 116.7 ± 17.5 (*p* = 0.6406). The mean differences and 95% confidence intervals confirmed no clinically relevant differences in postoperative blood glucose control between the two groups.

## Discussion

This study demonstrates that perineural dexamethasone significantly prolongs the duration of analgesia when added to the pericapsular nerve group (PENG) block in elderly patients undergoing total hip arthroplasty (THA). The findings align with existing research indicating that dexamethasone effectively extends nerve block duration by reducing inflammation and perineural oedema, as shown in studies involving femoral and sciatic nerve blocks [8–10]. The prolonged analgesic effect observed in this study led to a substantial delay in the need for rescue opioids, reducing overall opioid consumption. This is a critical advantage in elderly populations, who are particularly vulnerable to opioid-related complications such as respiratory depression, delirium, and gastrointestinal dysfunction [11–13].

The PENG block has gained recognition for its motor-sparing properties, making it particularly valuable in geriatric patients who require early mobilization [14, 15]. Early ambulation reduces the risk of postoperative complications, including venous thromboembolism (VTE) and prolonged hospitalization [16–18]. However, the main limitation of single-shot PENG blocks is their relatively short duration, with analgesia typically lasting around 9 h [19–21]. Adding perineural dexamethasone effectively extends this duration without compromising motor function, confirming its utility as an adjuvant in regional anaesthesia protocols [22, 23].

The role of dexamethasone was compared to other perineural adjuvants, such as dexmedetomidine, clonidine, and magnesium sulfate. While dexmedetomidine has been shown to prolong nerve blocks [24], it is often associated with dose-dependent sedation, bradycardia, and hypotension, making it less suitable for frail elderly patients [25]. Clonidine, another α2-adrenergic agonist, exhibits similar effects but with a higher risk of systemic hypotension [26]. Magnesium sulfate, though capable of extending block duration through NMDA receptor antagonism, has unpredictable pharmacokinetics and the potential to cause neuromuscular weakness [27]. Dexamethasone, in contrast, offers a more favorable safety profile, providing prolonged analgesia without significant haemodynamic or cognitive side effects [28].

A primary concern regarding perineural dexamethasone is its potential systemic effects, particularly hyperglycaemia [28]. However, this study found no significant differences in blood glucose levels between groups at 12, 24, and 48 h postoperatively. These findings align with prior research suggesting that perineural dexamethasone does not significantly impact glycaemic control compared to systemic administration [9, 29]. Furthermore, no cases of nerve injury were observed, reinforcing the safety of dexamethasone at the 4 mg dose used in this study. Previous studies have similarly reported no neurotoxic effects at low perineural dexamethasone doses, further supporting its safety in regional anaesthesia [9].

### Limitations

This study was conducted at a single orthopaedic centre, which may limit generalizability. The follow-up period was limited to 48 h, preventing an assessment of long-term outcomes such as chronic opioid use and functional recovery. The study did not compare perineural versus systemic (IV) dexamethasone, which remains an area of ongoing debate. While perineural dexamethasone showed clear benefits, some studies suggest IV administration may provide similar analgesic prolongation without potential concerns about perineural toxicity. Additionally, broader functional assessments were not included, including gait stability and quality-of-life metrics.

## Conclusion

This study supports the efficacy and safety of perineural dexamethasone as an adjuvant to the PENG block in elderly THA patients. The combination significantly prolongs analgesia, reduces opioid consumption, and preserves motor function without causing hyperglycaemia or nerve injury. Given its favorable safety profile compared to other adjuvants, perineural dexamethasone is a promising option for optimizing pain management in geriatric surgical patients. Future research should explore long-term functional outcomes, continuous perineural dexamethasone administration, and comparisons with systemic dexamethasone to refine perioperative analgesia strategies further. 

## Data Availability

No datasets were generated or analysed during the current study.
